# Scottish Pre-School Vision Screening – First 3 Years of National Data

**DOI:** 10.22599/bioj.138

**Published:** 2020-04-09

**Authors:** Lee Pentland, Sirjhun Patel

**Affiliations:** 1 Ninewells Hospital, NHS Tayside, GB

**Keywords:** vision screening, pre-school, amblyopia, POVS, See4School, orthoptist

## Abstract

**Introduction:**

Pre-school orthoptic vision screening (POVS) was implemented by the Scottish government and is a standardised assessment to promote early detection of visual problems in children. The target conditions are amblyopia, refractive errors and strabismus. We present the preliminary findings for the first three years of the screening program.

**Methods:**

The data from POVS was collected retrospectively. The data includes screening years 2013 to 2016 inclusive. Data was collected from each health board in Scotland. We report the coverage, referral rate, true positives and positive predictive values.

**Results:**

A total of 167,962 children were due to have vision screening over the 3 screening years included in this paper. This figure does not include the children that opted out of the eye test (mean opt-out rate 1.8%) and children that already attend the hospital eye service (mean already attend rate 3.1%). The POVS program had a mean coverage of 85.5%, ranging from 63.7% to 94.8% between health boards. Over the 3 year screening period, the mean referral rate was found to be 17.9%. The mean true positive rate was 88.9%, and the mean positive predictive value was 86.9%.

**Conclusion:**

The Scottish data set on pre-school orthoptic vision screening has shown excellent mean coverage. A consistently high true positive rate over the three screening years demonstrates it is a sensitive screening program, which is essential for the detection of visual problems in children.

## Introduction

Pre-school orthoptic vision screening (POVS) has been undertaken in all Scottish (mainland) health boards since 2012. This is a whole population, orthoptist-delivered screening with full audit data. We present an overview of some of the preliminary findings from the first three years of the national data collection from Scottish POVS.

In 2003, the fourth edition of the ‘Health For All Children’ report was published; this is more commonly known as the Hall 4 report (Hall and Elliman 2006). Guidance was published in 2005 on how best to apply the recommendations in Scotland (Howie 2005). It stated that “All children should be screened by an orthoptist in their pre-school year, between the ages of four and five years, removing the need for vision testing on school entry”. Prior to the publication of the Hall 4 report, provision of vision screening varied between health boards, with some health boards having no screening in place, whilst others had orthoptic vision screening in place for over 30 years. Following the publication of the Hall 4 report, the vision assessments that were offered as part of the universal pre-school child health program changed significantly. A *national* orthoptic delivered pre-school vision screening service was introduced. This replaced the 39-month Health Visitor assessment of vision and the Orthoptist vision screening in Primary 1, which had been implemented in only some health boards in pervious years.

The pre-school year in Scotland captures children between the ages of 3 years and 6 months and 5 years and 5 months, which is the ideal age for detecting the target conditions of amblyopia, strabismus and refractive errors (Williams 2009). By 2012, all mainland health boards in Scotland were collecting screening data on a national form, which was then uploaded to the national database at Information Services Division (ISD), within National Services for Scotland (NSS). The data presented in this article is from the first 3 complete years of pre-school orthoptic vision screening (2013/2014, 2014/2015 and 2015/2016).

## Methods

In Scotland, the pre-school year captures all children that will turn 4 between March 1st and the end of February the following year (e.g., the current 2018/2019 screening year will capture all children with a date of birth between 01/03/2014–28/02/2015, inclusive). These children are a similar age to reception age children in England and Wales. It should be noted, however, that these cohorts will not match in age exactly, as the school intake differs by six months in Scotland.

The cohort of eligible children is provided by each health board’s child health department. Therefore, any child registered with a GP in their health board area will be offered vision screening. Almost all of the orthoptic departments carry out the vision screening assessment in the children’s nursery setting. When possible, each health board’s orthoptic department will attempt to make repeat visits to the nurseries when/if required. Further vigilance is employed for children from deprived areas as it is recognised that they are more likely to require referral (O’Colmain et al. 2016; Williams et al. 2008). In addition, children from deprived areas are more likely to fail to attend offered appointments. If children are repeatedly absent from nursery or do not attend a nursery they will be offered an appointment at a local eye clinic.

The National Screening Committee (NSC) recommendation (UK NSC 2019) states “Children are asked to read the letters out from a particular distance until they can read no more”, which implies testing vision to threshold. The Scottish screening test includes an assessment of vision in each eye to threshold. Vision tests used include Keeler crowded LogMAR with a pass mark of 0.200, Sonksen crowded LogMAR with a pass mark of 0.100 (Salt et al. 2007) or crowded Kay Pictures with a pass mark of 0.100 (Saul and Taylor 2012; O’Boyle et al. 2017). If a child is unable to reliably match letters, the orthoptist will use crowded Kay Pictures. All health boards have the capacity to offer a recall, which is the opportunity to repeat the screening for those results thought to be doubtful on the day of testing.

The orthoptic delivered screening also includes cover test carried out for near and distance fixation, ocular motility assessment, convergence, assessment of binocularity using a 20^Δ^ prism reflex test and a pass/fail stereotest (1st plate of Frisby Stereotest or gross plates on TNO).

Any child who did not meet the visual acuity standard or was found to have an inter ocular difference (IOD) of three letters or more required referral. Children with a manifest strabismus, significant latent strabismus, ocular motility defects, binocular abnormalities (such as poor convergence and negative stereopsis) were referred at the discretion of the orthoptist.

There is a lack of evidence in the literature about what constitutes significant IOD in this age group of children. Due to the cohort of children being tested, there is potential for children as young as 3 years and 5 months being tested. The orthoptists wanted to ensure that children in the early stages of developing amblyopia were not being missed. It was decided that if an IOD of three letters or more was found this would require referral. For example, children with better than ‘pass mark’ vision could still potentially be referred if found to be at risk of developing amblyopia. The number of children referred solely from 3 letters of IOD was a total of 20 over the 3 year period (mean 0.07%, range 0.03%–0.11%) with a true positive rate of 50.5%. This referral criteria was found to be small enough to have negligible impact on the service.

The benefit of an orthoptic delivered screening service is that multiple tests can be carried out at the time of screening with only a small increase in test time, to ensure all amblyogenic risk factors are detected. An article published in the Ethics Journal of American Medical Association states, “Since few tests have both high sensitivity and high specificity, multiple tests are often used to aid in detection of disease” (Herman 2006). This incurs no additional cost to service provision in NHS Scotland. Furthermore it may reduce costs to the government in the form of educational support at school as it is well recognised that binocular vision anomalies are associated with visual symptoms when reading (Northway 2012; Northway 2013).

The vision screening program has an opt-out consent policy. Parent/carers have the option to opt their child out of the program.

## Results

The following tables provide an overview of the findings from the first three years of Scottish national data collection. Table 1 displays the number of children that were due to have their POVS. Over the 3 years, a total of 167,962 children were eligible for vision screening. This figure excludes children that have actively opted-out (mean withdrawn rate 1.8%, range 1.6%–2.2%) of the eye test and children that already attend the hospital eye service (mean 3.1%, range 2.2%–3.6%). The Scottish screening program had a mean coverage of 85.5% for the 3 years represented in this paper. This is slightly lower than coverage found in 2 audits of vision screening carried out by the British and Irish Orthoptic Society (BIOS) in the rest of the UK (range 89%–93%) (Carlton, Griffiths & Mazzone 2017; Griffiths, Carlton & Mazzone 2018). The data in the BIOS audits were limited by lack of feedback from participating sites. The 2015–2016 audit data includes analysis of results from 38 out of 204 sites (18.6%) and the 2016/2017 results from 42 out of 204 sites (20.5%). The results in our paper cover 100% of the mainland health boards in NHS Scotland.

**Table 1:** Coverage.

Screening Year	Number of Eligible Children*	Mean Percentage Screened	Range of Percentages per Health Board

2013/2014	56019	85.4%	63.7–94.8%
2014/2015	55919	87.0%	73.1–94.5%
2015/2016	56024	84.3%	66.5–92.8%

* The number of children eligible for vision screening is excluding children that opt-out of the service and children that already attend the hospital eye service.

It was noted the same health board had the lowest coverage for each of the screening years included in this paper. This health board had periods of staff shortages due to sickness/maternity leave, which led to patients being appointed at clinics rather than the orthoptists making nursery visits. If this board is excluded from the calculation, the mean coverage increases to 88%, similar that that of the BIOS audits.

Table 2 illustrates the referral rates for each screening years. The mean referral rate was found to be 17.9%. This was similar to previously published referral rates from other pre-school screening services. Hu and colleagues (2012) reported a referral rate of 17% and lower when compared with a study carried out by Buckley and Perkins (2010) (22%) that screened a similar age group of children.

**Table 2:** Referral Rate.

National Referral Rate			

Screening year	Number Referred	Percentage	Range per Health Board

2013/2014	8733	17.5%	13.7–25.6%
2014/2015	9191	18.1%	12.7–34.5%
2015/2016	9018	18.3%	8.9–36.8%

Each year some children are absent on the day of the screening at nursery or do not attend nursery. These children are therefore offered an appointment at a local clinic to have their vision screening carried out. Unfortunately, the attendance at these clinics is poor and there is a high number that “did not attend” (DNA). The mean DNA rate for the cohort for children due to be screened over the 3 screening years was 13.6% (range 12.3%–14.8%).

True positives were confirmed if significant refractive error was found. Significant refractive error was defined as hypermetropia or myopia ≥1.00 DS; ≥1.00 DC of astigmatism; anisometropia (either spherical or cylindrical) of ≥1.00 D. This was irrespective if glasses were prescribed at first visit. There is still no scientifically robust evidence as to prescription guidelines in children. Our cut off criteria was based on unpublished local audits of refractive errors that can cause reduced visual function if left uncorrected. Reassuringly, a review paper by Susan Leat (2011) provides evidence to support our criteria from multiple studies.

Other abnormalities such as significant latent squint, manifest squint, ocular motility defect, abnormality of binocular vision were also classed as positive referrals even if no treatment was required. In these cases, advice is given to parent/carers of symptoms to be aware of and, if relevant, the child monitored for deterioration of control of intermittent strabismus. A paper by Masqud and Medford (2015) found a mean false positive rate of 15.3% when comparing their study with 2 other orthoptic delivered services (13%, 13%, 20%). The mean false positive rate for our 3 years of screening was 13% (range; 11.2%–14.5%) which is similar to the false positive rate found by the studies in Masqud and Medford’s paper (2015). However, we need to bear in mind the variance of classification of false positive for these studies, for example significant myopia in Hu and colleagues’ (2012)^15^ paper was defined as “> +/–3.00 D”, which seems unreasonably strict especially with regards to myopia. Whilst Masqud and Medford (2015) acknowledge “no set protocols exist within the department for prescribing glasses and so were prescribed at the examiners discretion”.

Table 3 demonstrates the true positive and positive predictive value (PPV) for each screening year. The true positive rate of the program was found to be consistently high for the three screening years. The mean true positive rate was 88.9% (range 85.5%–88.8%). The screening programme has maintained a high PPV for the 3 years of national data collection with a mean PPV of 86.9% (range 85.8%–88.4%).

**Table 3:** True Positives and Positive Predictive Value.

Screening year	True Positives%	Positive Predictive Value%

2013/2014	85.5%	85.8%
2014/2015	86.6%	86.5%
2015/2016	88.8%	88.4%

## Discussion

It is evident from the Scottish data that the pre-school orthoptic vision screening service is providing excellent national coverage, high positive predictive values and true positive rates.

A previous study by Dent and Fieldsend (2015) suggested coverage is much better when screening is carried out in school compared to pre-school. They found the coverage at pre-school to be as low as 60% in contrast to 96% at school. This was similar to the health board in NHS Scotland that offered clinic appointments (65%). However, a higher mean coverage (88%) can be achieved when assessing children in their nursery setting. In Scotland, children receive a funded nursery place in their pre-school year (currently 16 hours per week, term time), and the uptake of this is very high. For example in 2013, 98% of eligible children were registered with a nursery (Furness 2013). Therefore, testing children in their pre-school year in Scotland is the optimum time to carry out the screening assessment not only for coverage but also for timely detection of our target conditions (Williams 2009).

Primary 1 children in Scotland are six months older than children in the equivalent year group in England. The age disparity between countries would result in later vision screening in Scotland if it were to be carried out in Primary 1. This may give rise to potential visual problems being identified six months later in comparison to England. A paper by Williams states “the most effective treatment to be ideally between 3 and 5 years of age, and under 7 years” (Williams 2009). Other studies also support early detection and treatment of amblyopia as best for visual outcomes (Hu et al. 2012; Koo, Gibert & Vanderveen 2017; Holmes et al. 2011). The Scottish programme of vision screening carried out in a child’s pre-school year allows early detection and treatment without compromising coverage and without an increase of false positives and ensures all children are starting school with the best possible vision. A study carried out by Bruce et al demonstrated the negative effect of non compliance with spectacle wear with children’s early literacy abilities (Bruce et al. 2018).

Masqud and Medforth (2015) stated “an orthoptic vision screening programme is the ‘gold-standard’, as shown by its excellent PPV and low re-test rates, and as such provides an excellent quality of service to children and parents”. Our findings from the vision screening taking place in NHS Scotland is providing evidence to support this statement of a gold standard service.

Our complimentary tests that check for amblyopia risk factors also have the added benefit of detecting binocular vision problems. Arguably, this is a controversial area, but there are publications discussing the detrimental effects on reading and learning abilities in children with uncorrected refractive error and undetected binocular vision problems (Northway 2012; Northway 2013; Leat 2011; Bruce et al. 2018; Christian et al. 2018; Scott et al. 2002). Undetected binocular vision anomalies can have a lasting life long impact (Northway and Dutton 2009).

There is no doubt testing vision, as a stand-alone screening tool, in an orthoptic-led service with a stringent training programme in place can also achieve good outcomes for detecting amblyopia (Garretty 2017). However, the service in Scotland is orthoptic delivered, and as such, the other tests are easily carried out as part of our service, and, therefore, do not result in any additional cost to the service that is provided. These tests do not cause any undue stress to the child and could potentially be more cost-effective in the long run for the child if unnecessary educational interventions can be avoided. The visual skills needed for reading are much more complex than identifying letters on a vision chart. A quality of life questionnaire is in the process of being designed with help from a research and development team in NHS Tayside. The aim is to gather information from parents and carers of children referred from pre-school screening for problems other than vision. It is hoped this will provide evidence the advice given at these appointments is invaluable and this shared decision making can help avoid future problems and anxieties.

## Limitations

There are still variabilities between health boards in the following areas: type of crowded Log MAR letter test used (Keeler and Sonksen), number of repeat visits to nurseries and the option to recall. However, all children in Scotland are offered an opportunity to have a standardised eye screening, which includes a crowded vision test and basic orthoptic assessment. This is important as there are no other vision screening episodes in childhood.

Unfortunately, some children may be missed when moving between health boards within the screening year. Regular communication between health boards and their child health department will help keep this number to a minimum.

## Conclusion

The current orthoptic delivered vision screening service in Scotland offers a comprehensive gold standard service with high true positives and high positive predictive values.

This comprehensive service detects amblyopia and its risk factors (refractive error and strabismus), which is the most common vision deficit in children in the UK (Tailor et al. 2016). It also detects muscle imbalance and binocular vision abnormalities, which if left undetected could potentially impact the child’s educational abilities and affect them into adulthood (Northway 2012; Northway 2013; Leat 2011; Christian et al. 2018; Scott et al. 2002; Northway and Dutton 2009).

Maintaining a high coverage is vital for capturing those children in more vulnerable backgrounds who are at higher risk of having visual defects (O’Colmain et al. 2016; Williams et al. 2008). Coverage appears to be lower in the health boards that offered clinic appointments rather than nursery visits. When possible, assessing children at nursery and repeat visit to the nurseries, especially in areas of deprivation, is vital for capturing the most vulnerable children in our communities.

The complimentary orthoptic screening tests help maintain an acceptable false positive rate and ensure full visual screening is offered to maximise every child’s potential when going to school. When discussing pre-school and school screening, we need to bear in mind the age group of children being discussed as this can vary from country to country.

Future work is needed to look at false negative data. This is likely to be low due to the orthoptic tests carried out at screening. For example, cover test at 33 cm on an accommodative target can pick up significant esophoria; even in the presence of normal vision, this could suggest underlying hypermetropia (Liu, Li & Li 2006; RNIB 2020).

As of September 2019, the POVS programme was renamed See4School. It is hoped this will promote better public awareness of the importance of vision screening and increase engagement with the screening program. The See4School program’s fundamental aim is early detection, referral and prompt treatment of visual defects. Vision screening can help reduce the prevalence of amblyopia (Solebo, Cumberland & Rahi 2014), which is vital for the prevention of avoidable blindness in future generations of an ageing population.

*Kay picture crowded test referred to in this article is the first edition crowded test*.

## References

[1] Bruce, A, Kelly, B, Chambers, B, Barrett, BT, Bloj, M, Bradbury, J and Sheldon, TA. 2018 The effect of adherence to spectacle wear on early developing literacy: A longitudinal study based in a large multiethnic city, Bradford, UK. BMJ open, 8: e021277 DOI: 10.1136/bmjopen-2017-021277 PMC6009541 29895654

[2] Buckley, CY and Perkins, AM. 2010 Comparison of visual outcomes at age 8 years in children detected by pre-school screening with those detected at reception screening. Br Ir Orthopti J, 7: 30–36. DOI: 10.22599/bioj.22

[3] Carlton, J, Griffiths, H and Mazzone, P. 2017 BIOS Screening Audit report 2015–2016. Figshare. Journal contribution [cited 2019 Jun 13]. DOI: 10.15131/shef.data.5532910.v1

[4] Christian, LW, Nandakumar, K, Hrynchak, PK and Irving, EL. 2018 Visual and binocular status in elementary school children with a reading problem. J Optom, 11(3): 160–166. DOI: 10.1016/j.optom.2017.09.003 29174394 PMC6039580

[5] Dent, M and Fieldsend, CS. 2015 A comparison of pre-school versus school-age orthoptic screening programmes in the North-East of England. Br Ir Orthopt J, 12: 16–19. DOI: 10.22599/bioj.90

[6] Furness, K. 2013 Summary Statistics for schools in Scotland, No. 4: 2013 Edition. 11 12 2013. Accessed 7 November 2019. Available at: https://www.gov.scot/publications/summary-statistics-schools-scotland-4-2013-edition/pages/22/.

[7] Garretty, T. 2017 Final Visual Outcomes and Treatment Received for Children Referred from a UK Primary School Visual Screening Program: A comparison of an orthoptic led program with orthoptic-delivered services. Strabismus, 25: 1–7. DOI: 10.1080/09273972.2017.1392988 29135316

[8] Griffiths, H, Carlton, J and Mazzone, P. 2018 BIOS Screening Audit report 2016–2017. Figshare. Journal contribution [cited 2019 Jun 13]. DOI: 10.15131/shef.data.6839813.v1

[9] Hall, DMB and Elliman, D. 2006 Health for All Children, 5th ed. Oxford University Press, Chapter 12. DOI: 10.1093/med/9780198570844.003.0012

[10] Herman, C. 2006 What Makes a Screening Exam “good”? Ethics Journal of The American Medical Association; Virtual Mentor, 8(1): 34–37. DOI: 10.1001/virtualmentor.2006.8.1.cprl1-0601 23232314

[11] Holmes, JM, Lazar, EL, Melia, BM, Astle, WF, Dagi, LR, Donahue, SP, Frazier, MG, Hertle, RW, Repka, MX, Quinn, GE and Weise, KK. 11 2011 Pediatric Eye Disease Investigator Group. Effect of age on response to amblyopia treatment in children. Arch Ophthalmol, 129(11): 1451–7. DOI: 10.1001/archophthalmol.2011.179 21746970 PMC3217111

[12] Howie, R. 2005 Health for All Children Draft Guidance on Implementation in Scotland: Analysis of Consultation Responses. Reid Howie Associates for the Scottish Executive. Available at: www.gov.scot/publications/health-children-4-guidance-implementation-scotland-2005/pages/5/.

[13] Hu, V, Starling, A, Baynham, SN, Wager, H and Shun-Shin, GA. 2012 Accuracy of referrals from an orthoptic vision screening program for 3- to 4-year-olds. JAAPOS, 16: 49–52. DOI: 10.1016/j.jaapos.2011.06.013 22237670

[14] Koo, EB, Gibert, AL and Vanderveen, DK. 2017 Treatment of Amblyopia and Amblyopia risk factors based on current evidence. Seminars in Opthalmology, 32: 1–7. DOI: 10.1080/08820538.2016.1228408 27748640

[15] Leat, SJ. 2011 To prescribe or not to prescribe? Guidelines for spectacle prescribing in infants and children. Clinical and Experimental Optometry, 94: 514–527. DOI: 10.1111/j.1444-0938.2011.00600.x 21722183

[16] Liu, X, Li, M and Li, Y. 2006 Spectacle correction of heterophoria in hyperopic amblyopic children. Journal of Zhejiang University. Science B, 7(11): 884–886. DOI: 10.1631/jzus.2006.B0884 17048302 PMC1635817

[17] Masqud, MA and Medforth, SD. 2015 Vision screening – referral to discharge. Outcomes from a routine vision screening programme. Br Ir Orthopti J, 12: 20–25. DOI: 10.22599/bioj.91

[18] Northway, N. 2012 Why do words jump? An exploration of visually symptomatic readers. British and Irish Orthoptic Journal, 9: 3–8. DOI: 10.22599/bioj.48

[19] Northway, N. 2013 Assessing reading and visual processing skills of young struggling readers: A pilot study. International Journal of Ophthalmic Practice, 3(5): 180–188. DOI: 10.12968/ijop.2012.3.5.180

[20] Northway, N and Dutton, GN. 2009 Undetected visual difficulties in adult literacies provision in Scotland. Learning Connections, Lifelong Learning Directorate, Scottish Government. Last accessed 27 January 2020. Available at: https://www.researchgate.net/publication/308119586_undetected_visual_difficulties_in_adult_literacy.

[21] O’Boyle, C, Chen, SI and Little, JA. 2017 Crowded letter and crowded picture logMAR acuity in children with amblyopia: A quantitative comparison. Br J Ophthalmol, 101: 457–461. DOI: 10.1136/bjophthalmol-2015-307677 27388249 PMC5583677

[22] O’Colmain, U, Low, L, Gilmour, C and MacEwen, CJ. 2016 Vision screening in children: A retrospective study of social and demographic factors with regards to visual outcomes. Br J Ophthalmol, 100(8): 1109–1113. DOI: 10.1136/bjophthalmol-2015-307206 26598576 PMC4975846

[23] RNIB Refractive errors (focussing problems). Last accessed 27 January 2020. Available at: https://www.rnib.org.uk/eye-health/eye-conditions/squint-childhood.

[24] Salt, AT, Wade, AM, Proffitt, R, Heavens, S and Sonksen, PM. 2007 The Sonksen logMAR Test of Visual Acuity: I. Testability and reliability. J AAPOS, 11(6): 589–596. DOI: 10.1016/j.jaapos.2007.04.018 17681815

[25] Saul, T and Taylor, K. 2012 Normative data for the crowded logMAR Kay’s pictures vision test in children. Br Ir Orthopti J, 9: 36–43. DOI: 10.22599/bioj.53

[26] Scott, L, McWhinnie, H, Taylor, L, Stevenson, N, Irons, P, Lewis, E, Evans, M, Evans, B and Wilkins, A. 2002 Coloured overlays in schools: Orthoptic and optometric findings. Ophthalmic and Physiological Optics, 22: 156–165. DOI: 10.1046/j.1475-1313.2002.00009.x 12014489

[27] Solebo, AL, Cumberland, PM and Rahi, JS. 2014 Whole-population vision screening in children aged 4–5 years to detect amblyopia. The Lancet, 385(9984): 2308–2319. DOI: 10.1016/S0140-6736(14)60522-5 25499167

[28] Tailor, V, Bossi, M, Greenwood, JA and Dahlmann-Noor, A. 2016 Childhood amblyopia: Current management and new trends. British Medical Bulletin, 119(1): 75–86. DOI: 10.1093/bmb/ldw030 27543498 PMC5862311

[29] UK NSC recommendation on Vision defects screening in children. Accessed 21 November 2019. https://legacyscreening.phe.org.uk/vision-child.

[30] Williams, C. 2009 Amblyopia. BMJ Clin Evid, 2009: 0709 DOI: 10.1055/s-0029-1217240 PMC2907781 21726480

[31] Williams, C, Northstone, K, Howard, M, Harvey, I, Harrad, RA and Sparrow, JM. 2008 Prevalence and risk factors for common vision problems in children: Data from the ALSPAC study. British Journal of Ophthalmology, 92: 959–964. DOI: 10.1136/bjo.2007.134700 18480306

